# Supplementary figures and images for: Ubiquitous Expression of mPolgmut Leads to the Accumulation of Cytotoxic CD8+ T Lymphocytes in Young Mice
Source: Life (Basel). 2025 Dec 5;15(12):1863. doi: 10.3390/life15121863 (PMC12735012; doi:10.3390/life15121863)

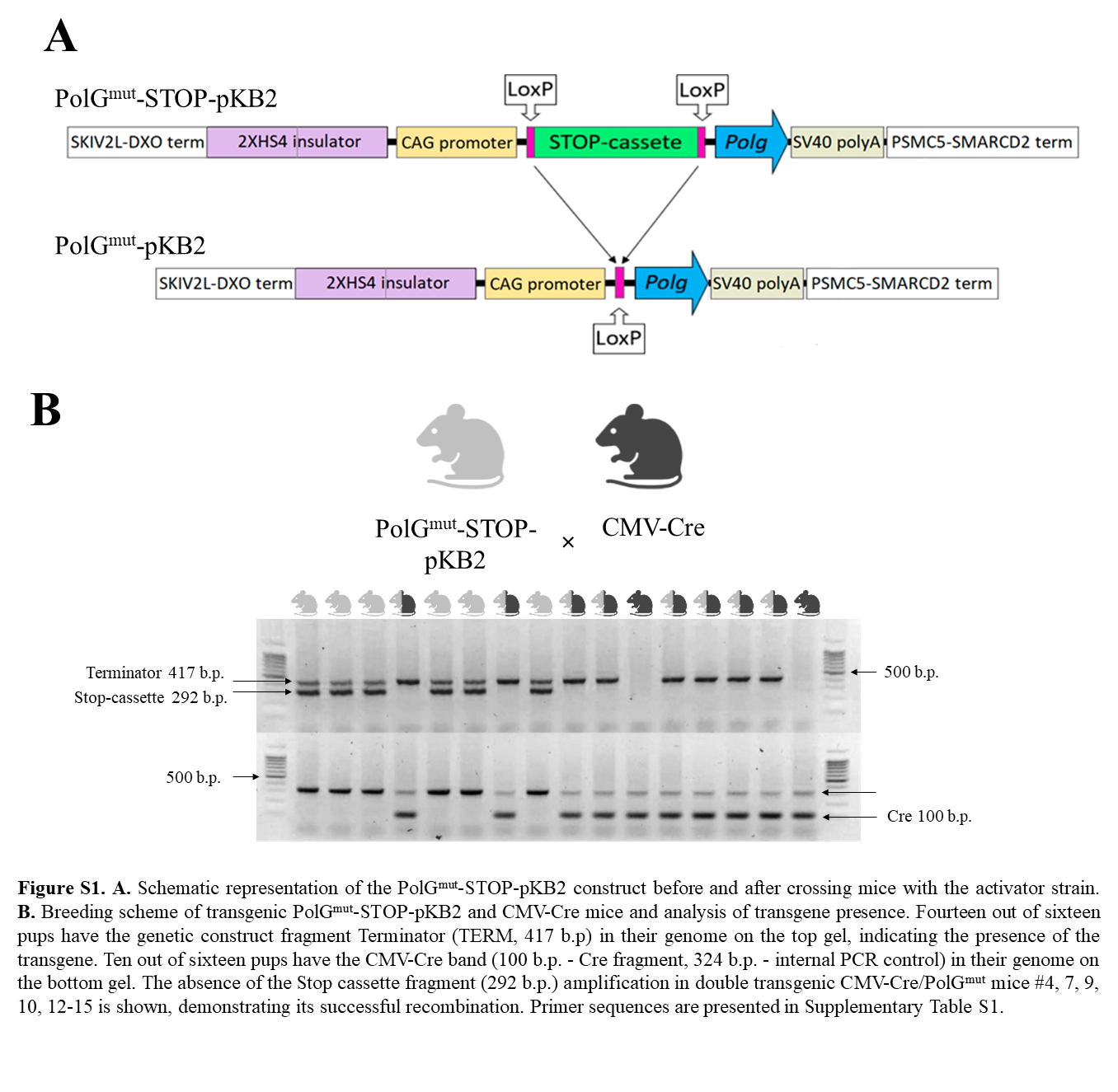

Supplement: Supplementary file 1 [file life-15-01863-s001.zip › Figure S1.tif]

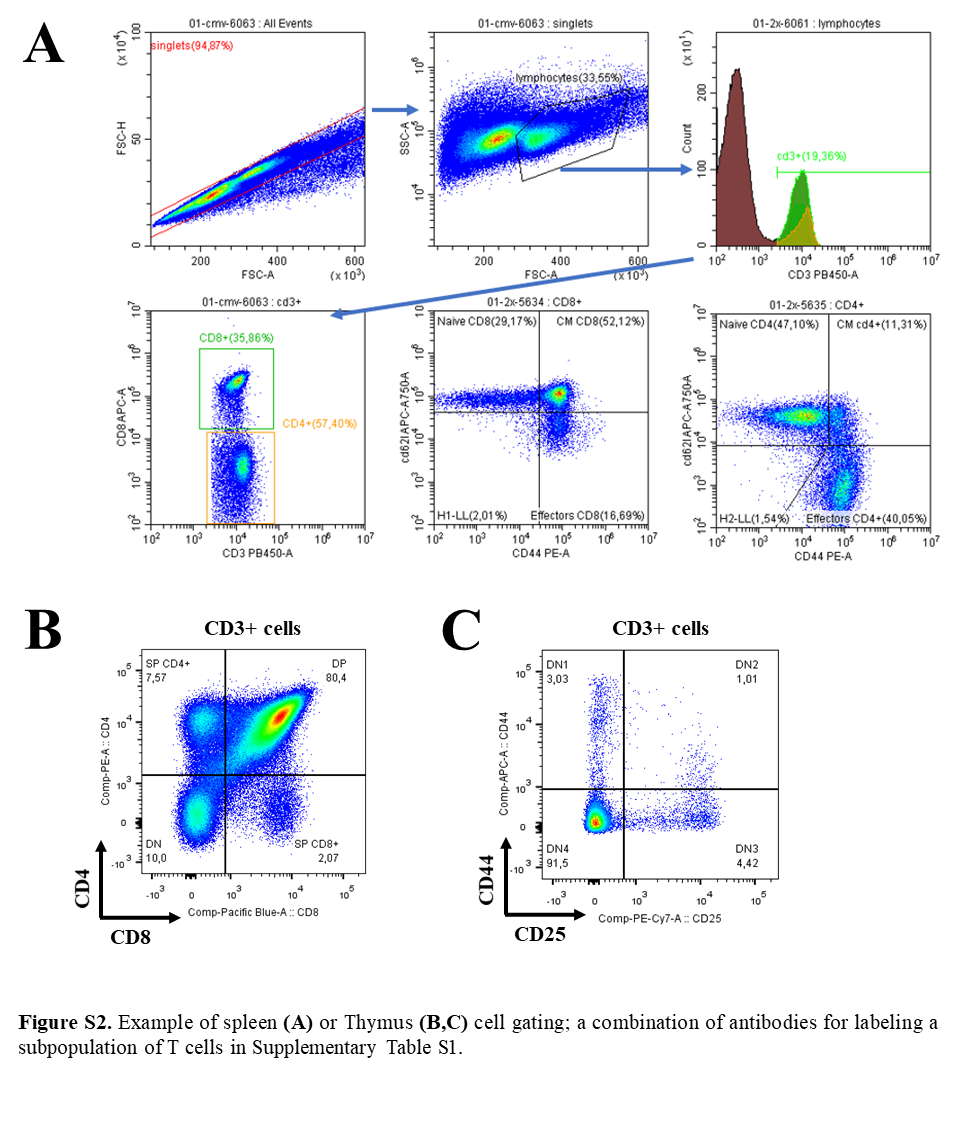

Supplement: Supplementary file 1 [file life-15-01863-s001.zip › Figure S2.tif]

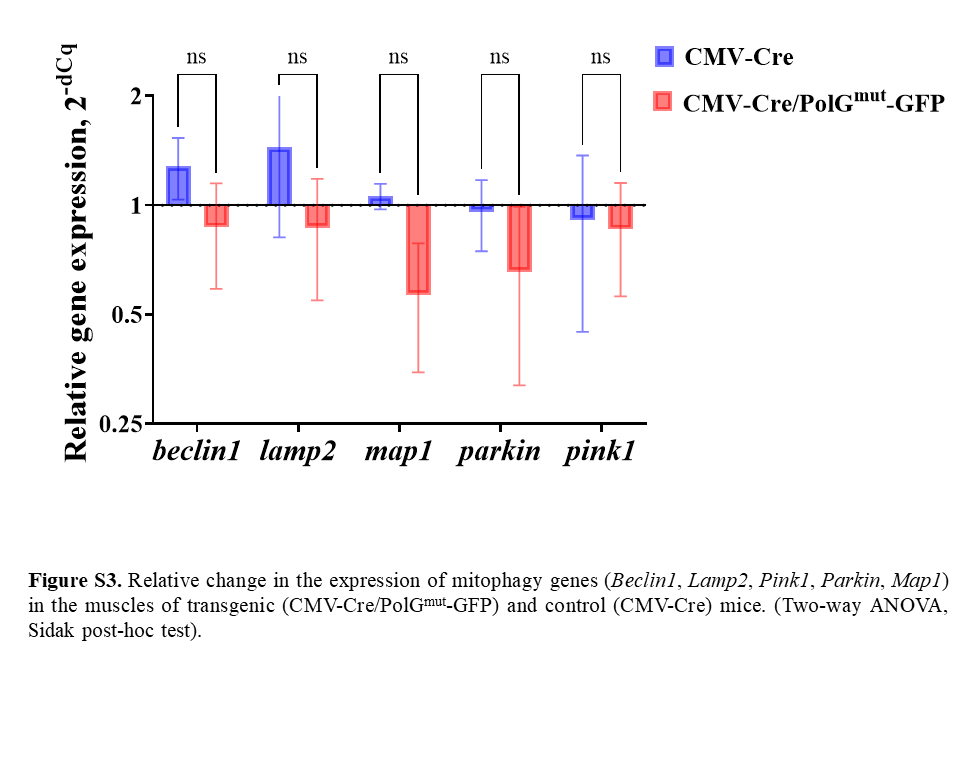

Supplement: Supplementary file 1 [file life-15-01863-s001.zip › Figure S3.tif]
